# Oleaginous yeasts respond differently to carbon sources present in lignocellulose hydrolysate

**DOI:** 10.1186/s13068-021-01974-2

**Published:** 2021-05-29

**Authors:** Jule Brandenburg, Johanna Blomqvist, Volha Shapaval, Achim Kohler, Sabine Sampels, Mats Sandgren, Volkmar Passoth

**Affiliations:** 1 Department of Molecular Sciences, Swedish University of Agricultural Sciences, BioCenter, Box 7015, 75007 Uppsala, Sweden; 2 Faculty of Science and Technology, Norwegian University of Life Sciences, P.O. Box 5003, 1432 Ås, Norway

**Keywords:** Oleaginous yeasts, Ascomycetes, Basidiomycetes, FTIR, Lipids, Lignocellulose, Xylose, Biofuels

## Abstract

**Background:**

Microbial oils, generated from lignocellulosic material, have great potential as renewable and sustainable alternatives to fossil-based fuels and chemicals. By unravelling the diversity of lipid accumulation physiology in different oleaginous yeasts grown on the various carbon sources present in lignocellulose hydrolysate (LH), new targets for optimisation of lipid accumulation can be identified. Monitoring lipid formation over time is essential for understanding lipid accumulation physiology. This study investigated lipid accumulation in a variety of oleaginous ascomycetous and basidiomycetous strains grown in glucose and xylose and followed lipid formation kinetics of selected strains in wheat straw hydrolysate (WSH).

**Results:**

Twenty-nine oleaginous yeast strains were tested for their ability to utilise glucose and xylose, the main sugars present in WSH. Evaluation of sugar consumption and lipid accumulation revealed marked differences in xylose utilisation capacity between the yeast strains, even between those belonging to the same species. Five different promising strains, belonging to the species *Lipomyces starkeyi*, *Rhodotorula glutinis*, *Rhodotorula babjevae* and *Rhodotorula toruloides*, were grown on undiluted wheat straw hydrolysate and lipid accumulation was followed over time, using Fourier transform-infrared (FTIR) spectroscopy. All five strains were able to grow on undiluted WSH and to accumulate lipids, but to different extents and with different productivities. *R. babjevae* DVBPG 8058 was the best-performing strain, accumulating 64.8% of cell dry weight (CDW) as lipids. It reached a culture density of 28 g/L CDW in batch cultivation, resulting in a lipid content of 18.1 g/L and yield of 0.24 g lipids per g carbon source. This strain formed lipids from the major carbon sources in hydrolysate, glucose, acetate and xylose. *R. glutinis* CBS 2367 also consumed these carbon sources, but when assimilating xylose it consumed intracellular lipids simultaneously. *Rhodotorula* strains contained a higher proportion of polyunsaturated fatty acids than the two tested *Lipomyces starkeyi* strains.

**Conclusions:**

There is considerable metabolic diversity among oleaginous yeasts, even between closely related species and strains, especially when converting xylose to biomass and lipids. Monitoring the kinetics of lipid accumulation and identifying the molecular basis of this diversity are keys to selecting suitable strains for high lipid production from lignocellulose.

**Supplementary Information:**

The online version contains supplementary material available at 10.1186/s13068-021-01974-2.

## Background

Limited fossil resources and increased use of first-generation biofuels demand exploration of renewable and sustainable alternatives. Currently used biofuels are mainly bioethanol and biodiesel [1]. Biodiesel is biodegradable, non-toxic and renewable, and can be used as a drop-in fuel together with fossil fuels, due to similar combustion properties [2, 3]. As a first-generation biofuel, it is produced mainly from oil-producing crops like rapeseed, soy or oil palm. This leads to direct competition between biofuels and food or feed production, or competition for agricultural land. Another problem is plantation of oil crops, such as oil palm, in rainforest areas [4, 5]. Lignocellulose is the most abundant biomass on earth [6] and has great potential as a raw material for microbial lipid production, decreasing the risk of competing with food production or land usage compared with first-generation substrates. When residues such as sawdust, straw, hemicellulose residues from the pulp and paper industry or crude glycerol are used, microbial lipid production can also add value to forestry and agriculture and improve the sustainability of these industries. Microbial oils can also be used for feed, food or other chemical applications [7–10].

However, lignocellulose is recalcitrant against degradation, and the conversion of lignocellulose requires a combination of thermochemical and enzymatic pretreatments to release the sugar monomers present in the biomass and make them available for the fermentation organism. Nevertheless, this pretreatment also releases inhibitors, such as acetic acid and other organic acids, furaldehydes and phenolics [11]. Acid steam explosion is a well-established method for thermochemical pretreatment [12] and we have used this method before for cultivating oleaginous yeasts. In spite of the released inhibitors, yeasts were able to assimilate this substrate and to form lipids on it [13].

Oleaginous microorganisms are defined by their ability to accumulate lipids in high amounts [14]. They are interesting for biofuel, feed or food production because their lipids have a similar fatty acid composition to vegetable oils [15, 16]. Oleaginous microorganisms are found among yeasts, filamentous fungi (e.g., *Mortierella alpina*, *Aspergillus oryzae*), bacteria (e.g., *Rhodococcus opacus*, *Bacillus megaterium*) and algae (e.g., *Chlorella zofingiensis*, *Arthrospira platensis*). Oleaginous yeasts have the highest known lipid accumulation rates among all organisms [17]. Lipid accumulation is widespread in yeasts and can be found in ascomycetes and basidiomycetes [18]. Lipid accumulation is triggered by nutrient limitation (nitrogen, sulphur, phosphorus) under a surplus of carbon [19–21].

Many oleaginous yeast species can convert a variety of carbon sources to lipids. These sources include glucose, xylose and organic acids, which are usually present in lignocellulosic hydrolysate. Numerous studies have examined conversion of lignocellulose hydrolysates to microbial lipids [7, 8]. System analyses show that this conversion process can have similar energy efficiency to bioethanol production from lignocellulose and can reduce CO_2_ emissions [22, 23]. However, the price of microbial lipid-derived diesel is still too high [24].

System analyses also show that rapid lipid production is crucial for obtaining a sustainable process, since aerobic cultivation requires much more energy input than the almost anaerobic process of ethanol production [22]. Identifying strains capable of rapidly producing lipids from lignocellulosic carbon sources would thus enable substantial progress towards sustainable microbial lipid production from lignocellulose. In a previous study, we found considerable differences between different oleaginous yeast species, with *Rhodotorula babjevae* having almost twice as high lipid productivity from wheat straw hydrolysate compared with *Lipomyces starkeyi* [25]. Identifying differences in the lipid accumulation physiology of different yeasts can facilitate identification of targets for manipulating yeast strains, enabling more efficient lipid accumulation on lignocellulose hydrolysate. One major path towards understanding lipid accumulation physiology is to monitor lipid accumulation kinetics over time. We recently developed spectroscopic methods that allow rapid quantification of intracellular lipids without pre-extraction [26–28].

The aim of the present study was to identify differences in lipid accumulation physiology between ascomycetous and basidiomycetous yeast species and strains grown in lignocellulose hydrolysate. A variety of strains belonging to the oleaginous genera *Lipomyces* and *Rhodotorula* were first tested on artificial cultivation media. Promising strains identified were then tested in wheat straw hydrolysate that was pretreated according to previous studies [13], to enable obtaining comparable results. The kinetics of carbon source consumption and lipid accumulation were monitored and analysed for patterns.

## Results

### Shake-flask growth test on model media

**Fig. 1:**
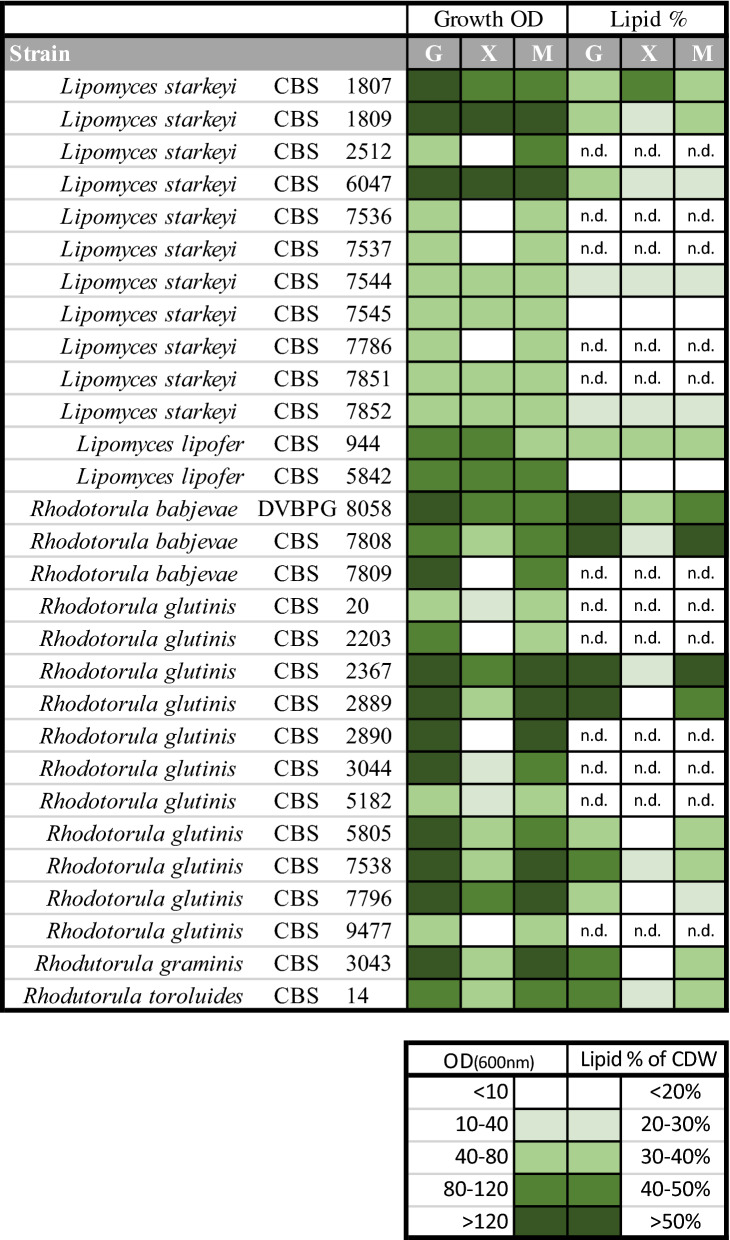
Growth and lipid content of *Rhodotorula* and *Lipomyces* strains grown in medium containing glucose (G), xylose (X) or a glucose/xylose mixture (M). The cultivations were done in shake flasks at 25 ºC, 130 rpm and performed in duplicates. Dark green represents strong growth and high lipid content, light green/white little or no growth and low lipid content. (nd) = not determined due to no or very poor growth. Exact values are given in Additional file 2

Twenty-nine different yeast strains (Additional file 1: Figure S1) were grown in shake flasks on glucose, xylose or a mixture of both. Growth performance and lipid accumulation are shown in Fig. 1.

In general, strains belonging to basidiomycetous species, including *Rhodotorula glutinis*, *R. babjevae*, *Rhodotorula graminis* and *Rhodotorula toruloides*, grew to higher final optical density than strains of the ascomycetous species, *Lipomyces starkeyi* and *L. lipofer*, when grown on glucose-containing media. In addition, the growth rate of basidiomycetous strains was higher than that of the ascomycetes. Surprisingly, many of the strains tested grew more slowly or not at all on xylose-based media. In particular, strains belonging to basidiomycetes (red yeasts) never reached the same culture density when grown on xylose compared with when grown on glucose. In total, seven strains showed very little growth on xylose (OD  ≤ 10) and four other strains showed only slow or little growth while utilising xylose (OD 10–40). Six of these strains belonged to *R. glutinis* (out of 10 strains of this species tested), one (out of three) to *R. babjevae* and four (out of 11) to *L. starkeyi* (Fig. 1). The four *Lipomyces* strains consumed little or none of the available xylose. In contrast, in the mixed culture broth these strains started consuming xylose when all available glucose had been consumed. However, the cultivation time of 144 h was insufficient for consumption of all the carbon sources. The seven *Rhodotorula* strains that did not perform well on xylose showed quite diverse growth behaviour. Some did not grow and did not consume xylose, some showed delayed and slow growth with little xylose consumption, and some strains grew slowly and consumed xylose, but did not attain substantial culture density. Even among the strains showing a tendency to assimilate xylose after glucose consumption in mixed culture broth, xylose consumption was still comparatively slow and was not completed within 144 h of cultivation time.

The 17 strains that showed good xylose consumption from the start were evaluated for lipid accumulation ability on glucose, xylose and the mixed substrate containing both sugars (Fig. 1). The intracellular lipid content of basidiomycetous strains when grown on glucose was 30–60% of cell dry weight (CDW), whereas on xylose the maximum intracellular lipid content was only 18–32% of CDW. Strains belonging to ascomycetes generally accumulated less intracellular lipids, especially when grown on glucose (13–37%), but achieved higher lipid content when grown on xylose (14–44%) (Additional File 2: Table S1).

Based on growth performance, five strains were selected for further investigations:

*Rhodotorula babjevae* DVBPG 8058, which grew to OD 135 on glucose and to OD 82 on xylose within 96 h. It had the highest lipid content observed on glucose (60.6%) and relatively high lipid content on xylose (31.9%).

*Rhodotorula glutinis*, and CBS 2367, which grew fast (maximum after 96 h) and to high density on glucose (OD 180) and xylose (OD 110 after 120 h). It had one of the highest measured intracellular lipid contents on glucose (58.6%) and relatively high lipid content when grown on xylose (28.7%).

*Rhodotorula toruloides* CBS 14, which showed good growth and lipid production, reaching OD 83 on glucose and OD 65 on xylose, with lipid content of 47.2% and 27.3%, respectively. It was selected for further investigation because it is regarded as one of the most promising oleaginous yeast strains.

*Lipomyces starkeyi* CBS 1807, which showed good overall growth performance, reaching OD 130 (after 96 h) on glucose and OD 105 (after 120 h) on xylose. It had the highest lipid content observed in this study when grown on xylose (44.5%). The lipid content on glucose was 37.5%.

*Lipomyces starkeyi* CBS 7544, which reached OD 65 on glucose and OD 62.5 on xylose, with lipid content of 22.7% and 23.9%, respectively.

### Cultivation on lignocellulose hydrolysate

These five selected strains were tested further for growth on undiluted hydrolysed wheat straw material (see “Methods”) in bioreactors under controlled conditions. As in previous tests (data not shown), the basidiomycetes stopped growing while carbon was still present in the hydrolysate. This effect was overcome by addition of ammonium phosphate, yeast nitrogen base (YNB, without amino acids and ammonium sulphate) and magnesium sulphate. Increasing the initial culture density from OD 1 to OD 5 overcame initial inhibitory effects of the hydrolysate for all strains. Acetic acid, which is inhibitory at low pH [29], was present in the media at a concentration of 5 g/L and was consumed by all strains within 48 h of cultivation. It thus acted as an extra carbon source rather than an inhibitor under these conditions (pH 6). All strains first consumed the glucose and acetic acid simultaneously, followed by utilisation of xylose. A preference of glucose over xylose as carbon source has been described before and is most probably due to glucose repression of xylose catabolism and/or competition for the same sugar transport protein [30, 31]. Simultaneous glucose/acetic acid consumption has been observed before [10], indicating that consumption of these carbon sources does not interfere with each other. Growth and lipid accumulation parameters are summarised in Table 1.

**Table 1:** Growth and lipid production by five oleaginous yeast strains on undiluted wheat straw hydrolysate

Strain	Cultivation time [h]	CDW [g/l]	Final lipid content [% of CDW]	Final lipid concentration [g/l]	Lipid yield [g/g]	Lipid production rate
*R. babjevae* DVBPG 8058	96	28.02^a^ (± 2.30)	64.83^a^ (± 2.99)	18.12^a^ (± 0.63)	0.24^a^ (± 0.01)	0.175^a^ (± 0.005)
*R. glutinis* CBS 2367	168	17.76^b^ (± 1.14)	12.11^b^ (± 1.10)	2.15^b^ (± 0.30)	0.03^b^ (± 0.01)	0.057^b^ (± 0.003)
*R. toruloides* CBS 14	91	29.82^a^ (± 0.25)	39.31^c^ (± 2.65)	11.72^c^ (± 0.64)	0.15^c^ (± 0.01)	0.176^a^ (± 0.009)
*L. starkeyi* CBS 1807	126	21.92^c^ (± 0.66)	33.15^d^ (± 2.11)	7.26^d^ (± 0.42)	0.09^d^ (± 0.01)	0.078^bc^ (± 0.012)
*L. starkeyi* CBS 7544	138	30.93^a^ (± 0.49)	38.48^ cd^ (± 1.67)	11.9^ cd^ (± 0.56)	0.16^c^ (± 0.01)	0.087^c^ (± 0.009)

Cultivations were performed in triplicate. Lipid yield was calculated as g lipids per g carbon source consumed, lipid content was determined gravimetrically by lipid extraction, lipid production rates were calculated based on lipid content measured by FTIR over time until the last time point before all carbon was consumed

Values in one column with different letters (a, b, c, d) are significantly different from each other (*p * < 0.05)

**Fig. 2:**
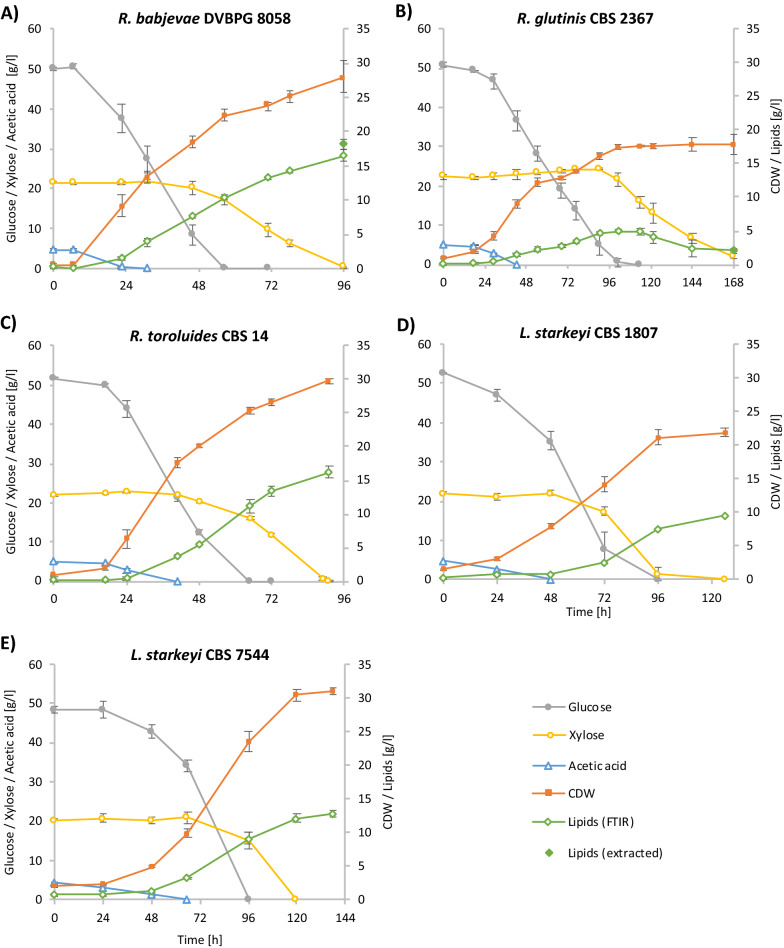
Lipid production, growth, sugar and acetic acid consumption of **a** *Rhodotorula* *babjevae* DVBPG 8058, **b** *Rhodotorula* *glutinis* CBS 2367, **c** *Rhodotorula toruloides* CBS 14, **d** *Lipomyces starkeyi* CBS 1807 and **e** *Lipomyces starkeyi* CBS 7544 grown in bioreactors containing wheat straw hydrolysate. All cultivations were performed in triplicates. Lipid production over time was determined by Fourier transform-infrared (FTIR) spectroscopy (calculated from biological triplicates, for *R. babjevae* DVBPG from duplicates). For *R.* *babjevae* DVBPG 8058 and *R. glutinis* CBS 2367 the end point values of lipid concentration were determined gravimetrically

*Rhodotorula babjevae* DVBPG 8058 was identified as the most efficient strain in converting wheat straw hydrolysate into intracellular lipids in this study. After 96 h of cultivation time, it achieved CDW of 28.02 g/L and an intracellular lipid content of 64.83% of CDW, resulting in a lipid concentration of 18.12 g/L. Calculated lipid yield per g carbon source was 0.24 (Table 1, Fig. 2a). The FTIR analysis [26] showed continuous lipid accumulation over time. Endpoint gravimetric lipid determination confirmed the intracellular fat content, with the analytical value being even higher than the value predicted from FTIR measurements.

Surprisingly, *R. glutinis* CBS 2367 did not accumulate high amounts of lipids until the end of cultivation, although it did so on the artificial medium. After 168 h of cultivation, all carbon source was consumed and a substantial CDW concentration density of 17.76 g/L was reached. This strain achieved a final lipid content of 12% of CDW. The FTIR measurements showed an increase in intracellular lipid content while consuming glucose, but showed lipid degradation while consuming xylose in the second phase of culture (Fig. 2b). Lipid extraction at the end of cultivation confirmed that in all three biological replicates lipid content decreased to a similar value (~ 12%). Lipid accumulation followed by degradation was confirmed in a second cultivation that included gravimetric lipid determination in the middle of cultivation in addition to the endpoint determination (Additional file 3: Figure S2).

*Rhodotorula toruloides* CBS 14 was able to grow on wheat straw hydrolysate and reached CDW of 29.82 g/L and a final intracellular lipid content of 39.31% of CDW. All carbon was consumed within 91 h of cultivation, with a yield of 0.15 g lipids/g carbon (Table 1).

*Lipomyces starkeyi* CBS 1807 reached 7.62 g/L CDW and accumulated 33.15% intracellular lipids after 120 h of cultivation, when almost all carbon source was finished. This was similar to the growth performance in artificial medium, but the lipid yield (0.09 g lipids/g consumed carbon source) was low compared with the basidiomycetous yeasts (Table 1), although in line with previous results for this strain [9, 25].

*Lipomyces starkeyi* CBS 7544 reached CDW of 30.9 g/L and intracellular lipid content of 38.48% of CDW. The lipid yield (0.16 g lipids/g consumed carbon source) was even higher than found for strain *L. starkeyi* CBS 1807, but 138 h of cultivation time were needed to finish all carbon sources (Table 1).

The FA-profiles (relative wt% of saturated fatty acids (SAT), monounsaturated fatty acids (MUFA) and polyunsaturated fatty acids (PUFA) of the five strains were predicted using the FTIR model described in Shapaval et al. [26] (Table 2). The ascomycetous strains had a significantly lower content of PUFA compared to the basidiomycetous strains. There were also significant differences in the amount of PUFA between the two *L. starkeyi* strains. The profile of *L. starkeyi* CBS1807 was similar to other studies with this strain [9] and to other strains of that species [32]. Among the *Rhodotorula* strains, *R. glutinis* CBS 2367 had the highest relative amount of PUFA. This could be due to the fact that the yeast consumed the storage lipids when glucose was finished, increasing the proportion of membrane lipids of the total lipids. This could explain the higher relative amount of PUFA compared to the other *Rhodotorula* strains investigated in this and other studies [33, 34]. The profile of *R. toruloides* CBS 14 was comparable to other studies using this strain [33, 35].

**Table 2:** Lipid profile determined by FTIR at the end of cultivation, or in case of *R. glutinis* 2367 24 hours before end of cultivations (endpoint determination was not available). Values are given in % of the total amount of fatty acids determined by FTIR

	SAT	MUFA	PUFA
Strain			
* R. babjevae* DVBPG 8058	28.87^a^ (± 0.35)	47.89^a^ (± 0.96)	23.34^a^ (± 0.67)
* R. glutinis* CBS 2367	36.51^ab^ (± 6.14)	23:95^b^ (± 5.74)	39.95^b^ (± 1.47)
* R. toruloides* CBS 14	30.47^a^ (± 0.16)	53.44^a^ (± 0.22)	16.09^c^ (± 0.07)
* L. starkeyi* CBS 1807	35.79^ab^ (± 0.88)	59.20^c^ (± 0.42)	5.02^d^ (± 0.49)
* L. starkeyi* CBS 7544	41.44^b^ (± 5.8)	57.99^c^ (± 5.81)	0.57^e^ (± 0.05)

Values in one column with different letters (a, b, c, d, e) are significantly different from each other (*p * < 0.05)

## Discussion

This study investigated the physiological diversity of 29 oleaginous yeast strains (ascomycetes and basidiomycetes) regarding their ability to produce lipids in model media containing the two major present in lignocellulosic hydrolysate sugars (glucose and xylose). Growth in wheat straw hydrolysate was also investigated. The results revealed considerable diversity in growth, sugar consumption and lipid formation rates, even among strains of the same species. The most striking metabolic diversity found was in ability to assimilate xylose and convert it to lipids. Seven of the 29 strains tested (three of 13 ascomycetes and four of 16 basidiomycetes strains), did not consume xylose when provided as the sole carbon source. Four other strains assimilated this sugar, but showed almost no growth. On the other hand, some strains with poor xylose assimilation started utilising xylose when grown in medium containing both glucose and xylose, after all glucose was finished. This marked variability in xylose assimilation was unexpected, since according to the taxonomic literature all strains tested should be able to grow on xylose as the sole carbon source [29, 36, 37].

Similar observations have been reported previously for some of the strains tested. In the ascomycetous yeast *L. lipofer* CBS 944, weak growth on xylose has been reported [29]. In the present study it showed slow growth, but still reached substantial culture density with complete xylose assimilation. *L. starkeyi* has been described as having good ability to utilise xylose [38], but several of the strains tested showed poor growth capacity on this sugar (CBS 2512, CBS 7536, CBS 7537, CBS 7786). Some basidiomycetous yeasts strains of *R.* *glutinis* have been reported to accumulate lipids when grown on xylose [39] or lignocellulose hydrolysate, including the hemicellulose fraction containing xylose [40]. However, the type strain of this species (CBS 20) is reported to show little growth on xylose as the sole carbon source [29], which was confirmed in this study. Several other strains of this species were found to show no growth at all on xylose as the single carbon source (e.g., CBS 9744 and CBS 2890). On the other hand, two *R. glutinis* strains (CBS 2367, CBS 7769) were able to grow on xylose and reach substantial culture densities. Similar cases were observed while cultivating *R. babjevae*, with two strains (CBS 7808 and DBGVP 5085) growing quite well on xylose, as reported earlier for this species [29]. However, one strain (CBS 7809) did not grow at all on xylose.

The observed variability in xylose assimilation among strains of different species belonging to different fungal phyla indicates that xylose can be a challenging substrate. For instance, the oleaginous yeast *Yarrowia lipolytica*, although possessing all genes required for xylose assimilation, is naturally unable to assimilate this sugar [41]. In *R. toruloides*, proteins involved in oxidative stress response are reported to be overproduced during growth on xylose compared with growth on glucose [35]. Xylose may induce stress responses in a variety of microorganisms, which would explain the difficulties in obtaining sustainable biofuel production from this sugar [31].

When following the kinetics of lipid accumulation over time, we observed another unexpected metabolic behaviour while utilising xylose. When *R. glutinis* CBS 2367 was cultivated in lignocellulose hydrolysate, it assimilated the xylose present when all glucose was exhausted. Simultaneously, intracellular lipids accumulated previously on glucose were degraded. In contrast, in strains DBVPG 5085 and CBS 14, belonging to the closely related species *R. babjevae* and *R. toruloides*, respectively, further lipid accumulation was observed when xylose was assimilated. This was also seen in the two *L. starkeyi* strains tested in detail. Moreover, when cultivated in model medium on a glucose/xylose mixture, high final lipid accumulation was observed in *R. glutinis* CBS 2367. These results show that it is necessary to monitor lipid accumulation kinetics over time, since this behaviour would have been overlooked when only running endpoint determination. In this study, monitoring was performed using our recently developed FTIR spectroscopy-based technique for intracellular lipid determination in oleaginous yeasts [26].

Overall, the results confirmed that xylose is a challenging substrate for many microorganisms. In *Saccharomyces cerevisiae* engineered for xylose fermentation, a starvation response on yeast containing all genes required for xylose assimilation has been reported [42]. In *R. toruloides*, induction of proteins involved in β-oxidation has been observed [35], which may also indicate a starvation response. Interestingly, feeding with xylose can result in lipid degradation even in higher eukaryotes [43]. It is possible that the magnitude of starvation response varies in different yeast strains, resulting in degradation of intracellular lipid reserves in some strains, but not in others. Additional stress factors present in lignocellulose hydrolysate may trigger the starvation response. More research on general gene expression and metabolic fluxes is required to understand the response of microbes to xylose and possible additional stress factors.

In general, growth in glucose was faster and more efficient for basidiomycetous strains, compared with ascomycetous strains. Microbial lipid production is performed in aerobic cultivation, which requires a considerably higher energy input per unit time than the more or less anaerobic ethanol production. Thus, shortening the cultivation time is an important factor to obtain sustainable microbial lipid production [23]. In this regard, *R. babjevae* DVBPG 8058 may represent a promising strain for future biotechnological lipid production, since it relatively rapidly reached an intracellular lipid content of almost 65% of CDW when growing on wheat straw hydrolysate. Similar high results have mainly been achieved previously in defined media [44] or under optimised cultivation conditions. Optimising culture and feeding conditions would presumably even enable a higher final lipid concentration than the 18.2 g/L seen in the present study. For instance, higher cell density during cultivation has been used in other studies with optimisation approaches [45]. In a previous study [25], *R. babjevae* DVBPG 8058 was also found to be superior to *L. starkeyi* CBS 1807 in converting wheat straw hydrolysate, from which furfural had been extracted, to lipids. However, in the present study the basidiomycetous yeasts needed extra nitrogen sources, but not the ascomycetous yeasts. To achieve a sustainable process, it is therefore necessary to identify cheap and abundant nitrogen sources. It could be possible to recirculate the nitrogen after lipid extraction for fermentation. Another approach could be to use whole yeast cells as a component of animal feed, e.g., in aquaculture [13]. This would mean using both lipids and nitrogen to create a high-value product. Therefore, the extra demand for nitrogen of basidiomycetous yeasts is probably not a serious barrier to biotechnological applications involving these yeasts, especially since they also produce other valuable compounds, such as carotenes [46, 47].

## Conclusions

This study revealed great diversity among oleaginous yeasts in lipid accumulation from the carbon sources present in lignocellulose hydrolysate. High variability was seen in both ascomycetous and basidiomycetous species. A great variety of responses was found especially when xylose was used as the carbon source. Some strains could not use xylose at all, others could only use it when provided in mixed substrate and several strains successfully converted it to biomass and lipids. FTIR spectroscopy-based lipid determination helped in identifying another pattern, of parallel xylose assimilation and lipid degradation, in one strain. Since these large differences were also found in strains and species that are closely related, i.e. have a similar genetic basis, there must be substantial differences in regulation of fluxes through the different pathways towards lipid synthesis. Studies identifying the mechanisms of regulation by monitoring lipid accumulation kinetics and broad comparative studies of genomics, transcriptomics, proteomics and metabolomics of a range of oleaginous strains can identify targets for molecular manipulation and theory-based selection of strains for sustainable, high-productivity biotechnological purposes.

## Methods

### Yeast strains and culture media

A set of 29 different oleaginous yeast strains were used for this study, of which 28 strains were obtained from CBS culture collection, Netherlands, and one from the Industrial Yeast Collection of Perugia (Italy), DVBPG (Fig. 1; Additional file 1: Figure S1).

All strains were stored at − 80 °C in 50% v/v glycerol and pre-grown on YPD agar slants (20 g/L glucose, 20 g/L peptone, 10 g/L yeast extract and 16 g/L agar) for 3 days at 25 °C. The plates were kept in a refrigerator and propagated monthly. Pre-cultures were grown in YPD medium (containing 20 g/L glucose, 20 g/L peptone and 10 g/L yeast extract) and cultivated for 2–3 days at 25 °C on an orbital shaker at 125 rpm.

The artificial cultivation medium contained: 1.7 g/L yeast nitrogen base (YNB) without ammonium sulphate and amino acids (Difco™, Becton Dickinson and Company, USA), 0.75 g/L yeast extract and 2 g/L ammonium sulphate. The pH was set to 6.0 and stabilised, using 0.2 M potassium phosphate buffer. As a carbon source, 70 g/L of either glucose or xylose, or a mixture of these two sugars (35 g/L glucose, 35 g/L xylose) was added to the medium.

### Hydrolysate preparation, steam explosion and enzymatic degradation

Wheat straw was hydrolysed by steam explosion and enzymatic degradation at the Department of Chemical Engineering, Lund University, Sweden, as previously described [13]. Briefly, wheat straw was soaked with 1% acetic acid overnight, and fluid removed by pressing. The acid soaked biomass was then steam exploded at 190 °C for 10 min in a 10 L steam pretreatment reactor. The liquid fraction (mainly hemicellulose) was separated from the solid fraction by pressing*.* The solid fraction was enzymatically hydrolysed. The hydrolysis was performed at 45 °C and pH 4.8. Cellic CTec3 enzyme cocktail (Novozyme A/S, Bagsværd, Denmark) was added at 10 FPU/g substrate. After enzymatic hydrolysis, the hydrolysate was centrifuged at 6000*g* and the liquid fraction was filtered in several steps to separate it from remaining solid residues (mainly lignin). The last filtration step was done using 0.45-µm bottle-top sterile filters (VWR Filter Upper Cup, Belgium). Before fermentation, the cellulose and hemicellulose fraction were mixed in a ratio of 60:40. The cellulose fraction contained approximately 87.3 g/L glucose, 22.2 g/L xylose and 3.8 g/L acetic acid and had a nitrogen content of 696.7 mg/L. The hemicellulose fraction contained approximately 8.9 g/L glucose, 34.7 g/L xylose and 7 g/L acetic acid and had a nitrogen content of 596.8 mg/L. After mixing, the hydrolysate had a proximate composition of 52.8 g/L glucose, 25.7 g/L xylose, 4.4 g/L acetic acid and 656.7 mg/L nitrogen.

### Cultivation

#### Screening in shake flasks

The screening experiment was performed in 250-mL baffled shake flasks containing 50 mL culture media. Each strain was grown on medium containing either glucose, xylose or the sugar mixture for 5–7 days in a table-top incubation shaker (Infors HT Ecotron, Switzerland) at 25 ºC and 130 rpm. Initial pH was 6. Pre-cultures were grown on YPD, washed twice with saline solution (i.e. the cells were centrifuged at 6000*g*, and re-suspended in 9 g/l NaCl solution, which was repeated one more time) and used for inoculation to an initial OD 1. All cultivations were performed in duplicate. Samples for OD and HPLC analyses were taken every 24 h, for lipid extraction from the pre-culture and at the end of cultivation and for CDW only at the end. Samples for FTIR spectroscopy were taken from pre-culture, at the end and for specific strains every 24 h.

#### Cultivation in bioreactors on hydrolysate

Five strains were cultivated in wheat straw hydrolysate in 500-mL bioreactors (Multifors 2, Switzerland) containing 350 mL hydrolysate. For cultivation of basidiomycetes, it was necessary to add 1.7 g/L YNB (without ammonium sulphate and amino acids, Difco™, Becton Dickinson and Company, USA), 0.5 g/L magnesium sulphate (Merck) and 2 g/L ammonium phosphate (Merck). Oxygen tension was set to 20% which was regulated by stirring speed (initially set to 200 rpm) with an air flow of 0.3 L/min. Temperature was set to 25 °C and polypropylene glycol 2000 was added manually as antifoaming agent, if needed. pH was set to 6 and controlled by 5 M NaOH and 3 M H_3_PO_4_ Bioreactors were inoculated to an initial OD 5. In preliminary experiments, we found that a higher initial cell density provides an improved tolerance against the inhibitors in the hydrolysate, shortens the lag phases and makes the cultivations more reproducible. This is in accordance with earlier findings on bioethanol production from lignocellulose hydrolysate [48]. Samples were collected at least once a day for CDW, HPLC analysis for sugar consumption and FTIR for lipid quantification. Samples for lipid analysis were taken from the pre-culture and at the end of fermentation.

### Analytical methods

Optical density was measured by a spectrometer (CO8000 Cell density meter, Nordic Biolabs) at a wavelength of 600 nm against distilled water.

Biomass was determined as CDW from 2 mL of culture broth. The samples were centrifuged at 17,000*g*, washed twice with deionised water and dried at 105 °C for 48 h, followed by weight measurement. CDW was determined in triplicate.

Sugar and acetic acid concentrations were determined by HPLC, using a Rezex-ROA-Organic Acid H^+^ 300 × 7.80 mm column and a refraction index detector as described previously [9].

Lipid extraction was performed by a solvent based phase separation method by Folch et al., using chloroform: methanol 2:1 [49] with some modifications. Main modifications were soaking 100 mg freeze-dried cells in 1 M HCL and incubating the samples at 75 °C for 1 h before extraction, as described in detail previously [9]. Total lipid content was determined by gravimetric measurement.

Lipid production over time was determined by Fourier transform-infrared (FTIR) spectroscopy (estimated from biological triplicates, for *R. babjevae* DVBPG from duplicates). For *R.* *babjevae* DVBPG 8058 and *R. glutinis* CBS 2367, the end point values for lipid concentration were determined gravimetrically.

Nitrogen in the hydrolysate was quantified by Kjeldahl analysis, using copper as a catalyst [50]. This analysis was performed at the Department of Animal Nutrition and Management, Swedish University of Agricultural Sciences. Standard deviation of the analysis was stated as maximum 0.9% of the total determined concentration.

#### Preparation of yeast biomass for FTIR spectroscopy

A 1–2 mL sample was taken from the cultivation, centrifuged at 17,000*g* and washed three times with 0.1% NaCl solution. After the last washing step, approximately 50 μL of cell suspension remained and was used for FTIR analysis [26].

#### High-throughput FTIR spectroscopy

Fourier transform infrared spectroscopic analysis of washed yeast biomass was performed using a High Throughput Screening eXTension (HTS-XT) unit coupled to a Vertex 70 FTIR spectrometer (both Bruker Optik, Germany) in transmission mode. For each cell suspension, 8 µL were transferred to an IR-light-transparent silicon 384-well microplate (Bruker Optik, Germany). Samples were dried at room temperature for 45 min before FTIR analysis. Spectra were recorded in the region between 4000 and 500 cm^−1^, with spectral resolution of 6 cm^−1^ and an aperture of 5.0 mm. For each spectrum, 64 scans were averaged [26]. FTIR spectra (4000–500 cm^−1^) were pre-processed using an approach resembling pre-processing strategies previously developed for lipids [51, 52]. The FTIR spectra were corrected by extended multiplicative scatter correction (EMSC) [53] with linear and quadratic components, followed by averaging of biological and technical replicates. Partial least squares regression (PLSR) was used to develop fatty acid profile prediction models by calibrating pre-processed FTIR spectra against gravimetric and gas chromatography (GC) reference data. The FTIR spectra were used as *X* variables (or predictors) and the gravimetric and GC fatty acid data as *Y* variables (or responses). The relative amount of SAT, MUFA and PUFA was predicted using the prediction models developed in Shapaval et al. [26].

#### Calculation of yields and rates

Lipid yields were calculated by dividing the total amount of formed lipids (i.e. difference of the lipid concentration at the endpoint of the fermentation and the lipid concentration at t_0_) by the amount of carbon source consumed at this time point. Lipid production rates were calculated by dividing the amount of lipids formed at the last the time point where carbon source was still present in the medium (i.e. before induction of an eventual starvation response) by the time required to form these lipids.

#### Statistical analysis

Results obtained for the five different strains tested in controlled conditions in bioreactors (see above) were analysed using single factor ANOVA with a significance level of *p * <  0.05. Pairwise comparison of strains was subsequently performed using a Tukey test, with a significance level of *p * <  0.05 [54]. Calculations were performed using Microsoft Excel 2016.

## Supplementary Information

**Additional file 1: Figure S1.** Photographs of the investigated strains on agar plates.

**Additional file 2: Table S1.** Screening of *Rhodotorula *and *Lipomyces *strains for growth on glucose, xylose and a mixture of both. All strains were grown on either a glucose- (G), xylose (X) or a glucose/xylose mixture (M) containing media. Growth was measured by OD- determination, Lipids were measured using FTIR spectroscopy. Lipid content was not determined (n.d.) for some strains due to exclusion of these strains from further investigation based on their poor growth performance. Experiments were performed in duplicates and average deviation is displayed, (*) only one replicate available.

**Additional file 3: Figure S2.** Cultivation of *R. glutinis* CBS 2367 grown on wheat straw hydrolysate. Intracellular lipid content was determined by lipid extraction at t0, 96 h and 192 h. It increased until all glucose was consumed and decreased during consumption of xylose.

## Data Availability

The datasets used and/or analysed during the current study are available from the corresponding author on reasonable request.
