# Supplementary material for: Oleaginous yeasts respond differently to carbon sources present in lignocellulose hydrolysate
Source: Biotechnol Biofuels. 2021 May 29;14:124. doi: 10.1186/s13068-021-01974-2 (PMC8164748; doi:10.1186/s13068-021-01974-2)
Supplement: Supplementary file 1 — Additional file 1: Figure S1. Photographs of the investigated strains on agar plates. [file 13068_2021_1974_MOESM1_ESM.pdf]

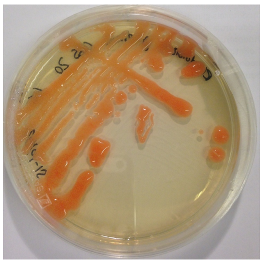
Rhodotorula glutinis CBS 20

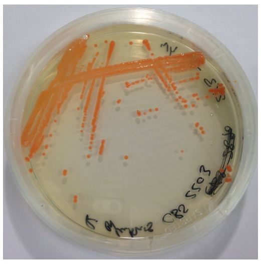
Rhodotorula glutinis CBS 2203

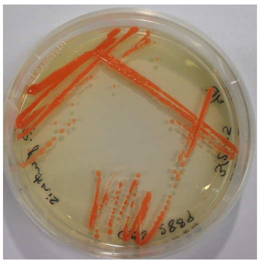
Rhodotorula glutinis CBS 2889

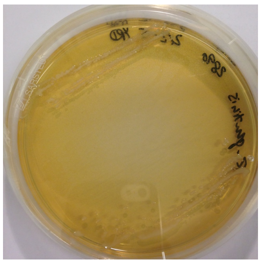
Rhodotorula glutinis CBS 2890

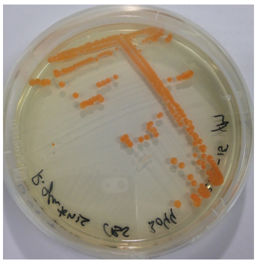
Rhodotorula glutinis CBS 3044

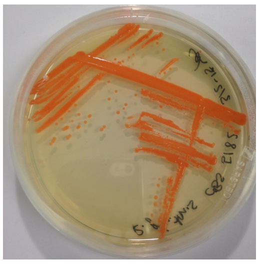
Rhodotorula glutinis CBS 5182

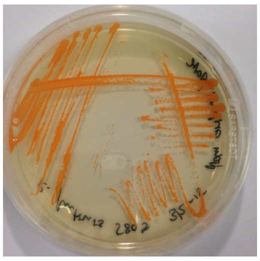
Rhodotorula glutinis CBS 5805

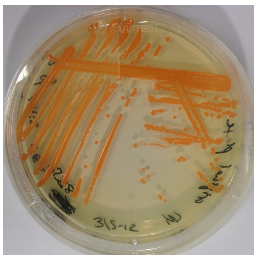
Rhodotorula glutinis CBS 7538

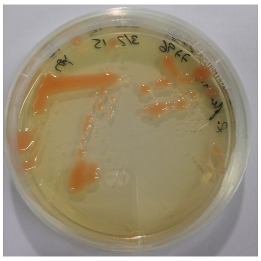
Rhodotorula glutinis CBS 7796

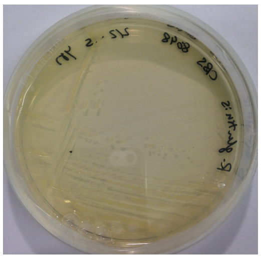
Rhodotorula glutinis CBS 8048

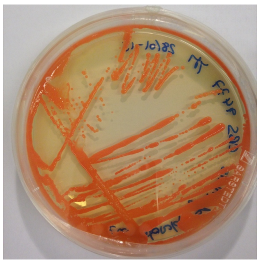
Rhodotorula glutinis CBS 9477

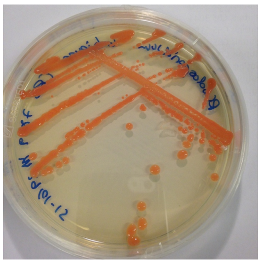
Rhodotorula babjevae CBS 7809

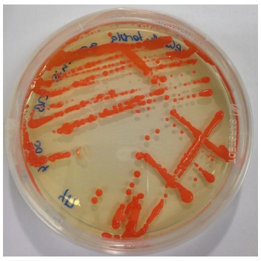
Rhodotorula graminis CBS 3043

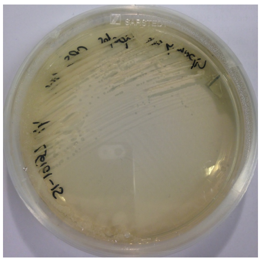
Lipomyces lipofer CBS 944

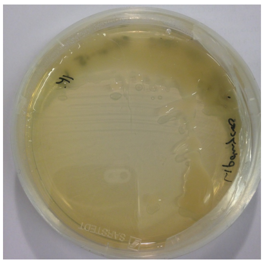
Lipomyces lipofer CBS 5842

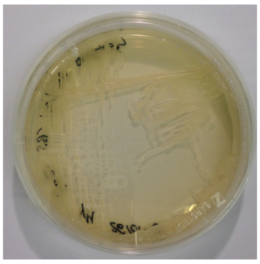
Lipomyces starkeyi CBS 1807

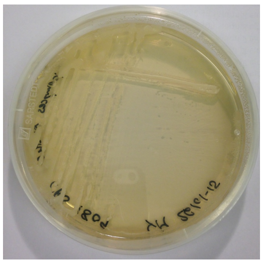
Lipomyces starkeyi CBS 1809

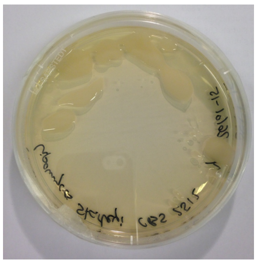
Lipomyces starkeyi CBS 2512

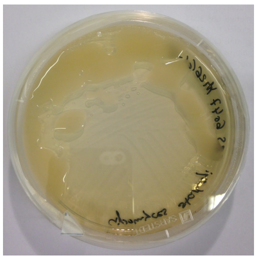
Lipomyces starkeyi CBS 6047

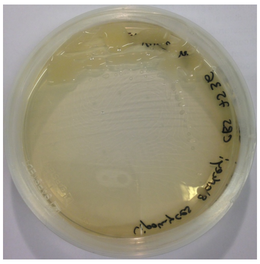
Lipomyces starkeyi CBS 7536

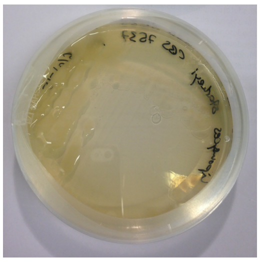
Lipomyces starkeyi CBS 7537

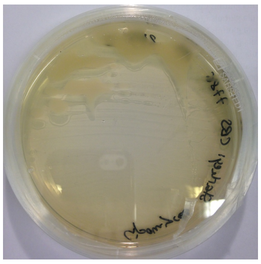
Lipomyces starkeyi CBS 7786

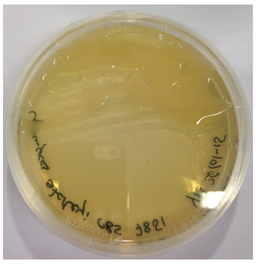
Lipomyces starkeyi CBS 7851

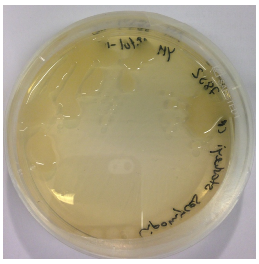
Lipomyces starkeyi CBS 7852
