# Supplementary material for: Oleaginous yeasts respond differently to carbon sources present in lignocellulose hydrolysate
Source: Biotechnol Biofuels. 2021 May 29;14:124. doi: 10.1186/s13068-021-01974-2 (PMC8164748; doi:10.1186/s13068-021-01974-2)
Supplement: Supplementary file 2 — Additional file 2: Table S1. Screening of Rhodotorula and Lipomyces strains for growth on glucose, xylose and a mixture of both. All strains were grown on either a glucose- (G), xylose (X) or a glucose/xylose mixture (M) containing media. Growth was measured by OD- determination, Lipids were measured using FTIR spectroscopy. Lipid content was not determined (n.d.) for some strains due to exclusion of these strains from further investigation based on their poor growth performance. Experiments were performed in duplicates and average deviation is displayed, (*) only one replicate available. [file 13068_2021_1974_MOESM2_ESM.pdf]

Strain			Growth OD G	±	Growth OD X	±	Growth OD M	±	Lipid % G	±	Lipid % X	±	Lipid % M	±
Lipomyces starkeyi	CBS	1807	130	20	105	2.5	105	2.5	37.06	0.71	44.45	0.89	35.89	0.59
Lipomyces starkeyi	CBS	1809	120	0	120	0	130	0	30.14	0.33	29.92	0.03	30.86	0.02
Lipomyces starkeyi	CBS	2512	76	0	9	0	95	2.5	n.d.		n.d.		n.d.	
Lipomyces starkeyi	CBS	6047	155	5	160	5	140	0	30.08	1.44	23.88	0.06	24.08	0.97
Lipomyces starkeyi	CBS	7536	68	0	4.7	0.05	64	2	n.d.		n.d.		n.d.	
Lipomyces starkeyi	CBS	7537	62	2	1.1	0	67	4.5	n.d.		n.d.		n.d.	
Lipomyces starkeyi	CBS	7544	65	1	62.5	0.75	68	0	22.71	0.91	23.93	0.06	27.38	0.32
Lipomyces starkeyi	CBS	7545	64	0	64	2	55	0.5	13.64	2.55	19.53	0.84	16.75	0.25
Lipomyces starkeyi	CBS	7786	53	1	6.6	0.2	58	1	n.d.		n.d.		n.d.	
Lipomyces starkeyi	CBS	7851	50.5	0.5	48.5	0.75	52.5	0.75	n.d.		n.d.		n.d.	
Lipomyces starkeyi	CBS	7852	56	6	62	1	56	0	24.89	0.19	25.78	0.05	29.44	0.82
Lipomyces lipofer	CBS	944	84	2	85	1.5	79	1.5	32.48	2.55	35.54	1.36	33.99	1.59
Lipomyces lipofer	CBS	5842	83	3	84	2	83	0.5	15.06	0.37	14.01	0.49	11.79	0.15
Rhodotorula babjevae	DVBPG	5805	135	15	82	1	88	5	60.64	2.17	31.86	0.31	48.72	1.35
Rhodotorula babjevae	CBS	7808	110	10	47	2	96	1	54.18	0.41	25.43	3.02	50.54	0.37
Rhodotorula babjevae	CBS	7809	150	0	2.1	0	105	5.5	n.d.		n.d.		n.d.	
Rhodotorula glutinis	CBS	20	56	2	33.5	0.75	51	2.5	n.d.		n.d.		n.d.	
Rhodotorula glutinis	CBS	2203	81	1	1.65	0.075	64	0	n.d.		n.d.		n.d.	
Rhodotorula glutinis	CBS	2367	180	0	110	*	175	2.5	58.59	0.50	28.65	*	55.17	0.07
Rhodotorula glutinis	CBS	2889	170	10	60	*	150	0	50.11	*	17.17	*	45.50	0.05
Rhodotorula glutinis	CBS	2890	170	10	10	0	160	0	n.d.		n.d.		n.d.	
Rhodotorula glutinis	CBS	3044	125	5	24	0.5	91	0.5	n.d.		n.d.		n.d.	
Rhodotorula glutinis	CBS	5182	79	3	33.5	0.75	77	0.5	n.d.		n.d.		n.d.	
Rhodotorula glutinis	CBS	5805	165	15	47	1	100	0	38.82	1.27	19.64	1.28	32.48	0.11
Rhodotorula glutinis	CBS	7538	130	0	72	2	150	5	43.21	1.97	23.54	0.90	39.28	0.71
Rhodotorula glutinis	CBS	7796	150	0	96	0	140	0	30.20	*	18.96	0.54	23.03	0.32
Rhodotorula glutinis	CBS	9477	61	1	5.75	0.825	58	2	n.d.		n.d.		n.d.	
Rhodotorula graminis	CBS	3043	165	5	53	0.5	140	0	44.14	1.92	18.27	0.63	33.97	1.36
Rhodotorula toruloides	CBS	14	83	3	65	2.5	82	0	47.17	2.02	27.32	1.38	37.62	0.33
