# Supplementary figures and images for: Oleaginous yeasts respond differently to carbon sources present in lignocellulose hydrolysate
Source: Biotechnol Biofuels. 2021 May 29;14:124. doi: 10.1186/s13068-021-01974-2 (PMC8164748; doi:10.1186/s13068-021-01974-2)

*R. glutinis* CBS 2367

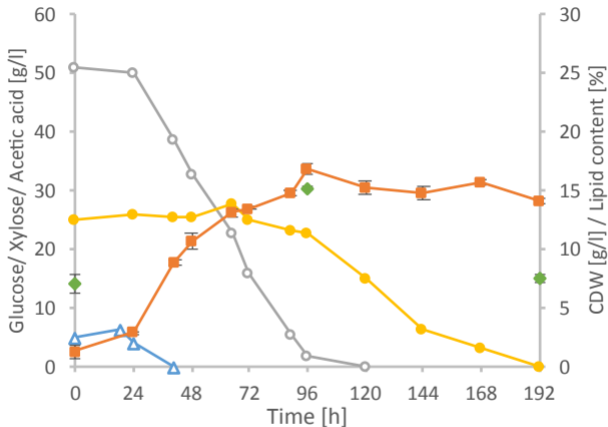

—○— Glucose      —●— Xylose      —△— Acetic acid  
—■— CDW      ◆ Lipid content

Supplement: Supplementary file 3 — Additional file 3: Figure S2. Cultivation of R. glutinis CBS 2367 grown on wheat straw hydrolysate. Intracellular lipid content was determined by lipid extraction at t0, 96 h and 192 h. It increased until all glucose was consumed and decreased during consumption of xylose. [file 13068_2021_1974_MOESM3_ESM.pdf]
